# A parallel accumulator model accounts for decision randomness when deciding on risky prospects with different expected value

**DOI:** 10.1371/journal.pone.0233761

**Published:** 2020-07-23

**Authors:** Jonathon R. Howlett, Martin P. Paulus

**Affiliations:** 1 Department of Psychiatry, University of California San Diego, La Jolla, California, United States of America; 2 Laureate Institute for Brain Research, Tulsa, Oklahoma, United States of America; University of Sheffield, UNITED KINGDOM

## Abstract

In decision-making situations individuals rarely have complete information available to select the best option and often show decisional randomness, i.e. given the same amount of knowledge individuals choose different options at different times. Dysfunctional processes resulting in altered decisional randomness can be considered a target process for psychiatric disorders, yet these processes remain poorly understood. Advances in computational modeling of decision-making offer a potential explanation for decisional randomness by positing that decisions are implemented in the brain through accumulation of noisy evidence, causing a generally less preferred option to be chosen at times by chance. One such model, the linear ballistic accumulator (LBA), assumes that individuals accumulate information for each option independently over time and that the first option to reach a threshold will be selected. To investigate the mechanisms of decisional randomness, we applied the LBA to a decision-making task in which risk and expected value (EV) were explicitly signaled prior to making a choice, and estimated separate drift rates for each of the four task stimuli (representing high and low EV and high and low risk). We then used the fitted LBA parameters to predict subject response rates on held-out trials for each of the 6 possible stimulus pairs. We found that choices predicted by LBA were correlated with actual choices across subjects for all stimulus pairs. Taken together, these findings suggest that sequential sampling models can account for decisional randomness on an explicit probabilistic task, which may have implications for understanding decision-making in healthy individuals and in psychiatric populations.

## Introduction

Although a long tradition in economics has established principles of rational decision-making given a set of preferences, abundant evidence has emerged that humans often make irrational choices, giving rise to the discipline of behavioral economics [1]. The field of neuroeconomics has also emerged with the goal of characterizing the neural underpinnings of (both rational and irrational) human choices [2]. Given evidence of disordered decision-making in individuals with psychiatric disorders (such as mood and anxiety disorders) [3], the behavioral and neural aspects of rational and irrational choices have important implications for assessment and treatment of mental health problems. Recently, fine-grained computational models of decision-making have been applied to more specifically delineate processing dysfunction in psychiatric disorders at a mechanistic level [4].

One complex aspect of human decision-making is the apparent incorporation of randomness into choices in value-based decision paradigms. In general, economic models predict that individuals always (deterministically) choose the option with the highest expected value. However, research has shown that expected values are mapped proportionally onto option probabilities and that the option with the highest expected value will be chosen most often but not always. This probabilistic (i.e. random) aspect of value-based choice is frequently modeled descriptively using probabilistic decision policies such as a softmax decision rule, which includes a parameter that controls the degree of decisional randomness (ranging from completely deterministic to completely random) [5]. Under certain circumstances, such apparent randomness can represent a rational process, such as when the environment might be changing [6] or when valuable information can be gained by exploring an apparently lower value choice [7]. However, this behavior can be observed even in the absence of a rational justification, in which case it has been termed random (as opposed to directed) exploration [8].

Advances in computational modeling may go beyond descriptive modeling and shed light on the fundamental mechanistic processes underlying randomness in value-based decisions. These choices have recently been explained in terms of sequential sampling models, in which evidence in favor of each alternative accumulates in real time, and a decision is reached once the evidence in favor of one alternative reaches a certain threshold. One example of a sequential sampling model is the drift diffusion model (DDM), in which noisy evidence is accumulated over time toward one of two decision thresholds, with the average rate of accumulation being determined by a drift rate parameter, but with noise in the accumulation process leading to variability in responses and response times [9]. The DDM accounts for the speed accuracy tradeoff during decision making via the degree of separation of the decision boundaries, and is a parsimonious framework that incorporates both responses and response times in decision-making. DDM has recently been applied to reinforcement learning tasks, in which the values of alternatives must be learned from experience over time, and these estimated values are then incorporated to determine which option is chosen. While this decision process has typically been modeled in a descriptive fashion using the softmax rule (as discussed above), the DDM has been proposed as a specific mechanism by which learned values are incorporated into the decision process [10]. In this hybrid reinforcement-learning/DDM approach, the difference in learned values between options determine the drift rate for the DDM, thus establishing the probability of each response and the reaction time distribution. Because of the noisy nature of the DDM process, the choice is not deterministic, and the lower-valued option will sometimes be chosen (as is observed empirically). Thus, by incorporating reaction times in addition to responses, a sequential sampling model can potentially illuminate the processing dynamics giving rise to randomness in decision-making.

An alternative to DDM is the linear ballistic accumulator (LBA) model, which differs in that there is a separate, independent accumulator representing each alternative, each with its own drift rate, and these parallel accumulators race against each other to determine which alternative is chosen [11]. These two models share several attributes, including the noisy accumulation of evidence with a drift rate parameter representing the rate of accumulation, and a decision threshold parameter controlling the speed accuracy tradeoff. LBA differs from DDM in that it posits a separate, parallel accumulation process for each option, rather than a single accumulation process toward one of two decision boundaries. Another difference is that LBA omits within-trial randomness, and instead explains outcome randomness in terms of variability in drift rates and starting points for accumulation from trial to trial. The parameters of the LBA include: (1) a drift rate, which represents the average rate of accumulation, (2) an upper decision threshold, with lower values representing faster but less accurate choices and higher values representing slower and more accurate choices, (3) a maximum starting-point (in the LBA, the starting-point for evidence accumulation is randomized based on a uniform distribution from 0 to the maximum starting-point), and (4) a non-decision time, which represents the time to register a stimulus prior to the decision process and time to prepare and execute a response once a decision is reached.

Decisional randomness in the context of learning can serve a rational function by allowing for exploration of alternatives that appear to have a low value but may in fact have a high value. Less is known about decisional randomness in the context of explicit decision-making tasks without a learning component, which may have implications for dysfunctional decision-making in clinical populations. In particular, dysfunctional decision-making in individuals with psychiatric disorders may be due either to impaired learning of the value of different options, or in impaired incorporation of this information into a decision (i.e. impaired sequential sampling), and these different impairments may have different underlying neural mechanisms with implications for assessment and treatment. In order to investigate whether an evidence accumulation model could account for decision randomness in this context, we applied an LBA to a task in which the expected value and risk of each alternative were signaled to subjects prior to their decision. In a previous investigation using a similar task [12], we found that that the concavity of the subjective utility curve predicted risk preferences (in agreement with economic expected utility theory [13]) and that trait anxiety was not associated with risk preferences or with sensitivity to counterfactual outcomes. In the current study, using a similar task, we sought to replicate these results in a separate sample, while extending our prior work by applying the LBA model to account for value- and risk-based choices. We hypothesized that LBA parameters could predict subject choices, thus providing evidence that a parallel accumulation model could explain decision randomness on an explicit probabilistic task.

## Materials and methods

### Participants

Forty-four college students (age: 19.34 ± 2.28 years; 26 females and 18 males) participated in this study. Subjects were recruited from San Diego State University through an online system as part of Psychology 101 class during the spring of 2013. They were contacted and scheduled for an experimental session during winter quarter 2013. The study was approved by the Human Research Protections Program at San Diego State University. All participants provided written informed consent, and were compensated $25 and 2.5 course credits for completing the study.

Prior to completing the experimental task, participants completed the State-Trait Anxiety Inventory, Trait scale (STAI-T) to assess trait anxiety [14].

### Decision-making task

Subjects completed a decision-making task [12] consisting of 4 blocks with 24 trials per block. On each trial, subjects were asked to choose one of two gambles. They were asked to imagine they were choosing between two different random drawings with different numbers of chips worth 0, 20, or 40 points (subjects played for virtual points rather than real money). For each of the two options, subjects were shown the number of each type of chip (out of a total of 100). Subjects were thereby shown the value and probability of each possible outcome. There were four different stimuli (i.e. four different drawings) used in the task with different risk and expected value (EV) profiles: high risk/high EV, high risk/low EV, low risk/high EV, and low risk/low EV. High risk gambles had variance 384 (with larger numbers of 0-point and 40-point chips), while low risk gambles had variance 96 (with larger number of 20-point chips). High EV gambles had EV of 24 points, while low EV gambles had EV of 20 points. With 4 different stimuli, there were 6 different possible pairs of stimuli, each of which was encountered 16 times during the course of the task (subjects were never asked to choose between two identical stimuli). After making a selection, subjects were shown the outcome of their choice (either 0, 20, or 40 points, which was determined by a random number generator in accordance with the stated probabilities). Half of blocks were “Counterfactual Feedback” blocks, in which subjects were shown what they would have received if they had made the opposite choice. The other half were “No Counterfactual Feedback” blocks, in which subjects were not shown the outcome of the opposite choice. The order of “Counterfactual Feedback” and “No Counterfactual Feedback” blocks was counterbalanced between subjects. After receiving feedback, subjects were asked to indicate their level of satisfaction with their choice on a visual analog scale.

### Behavioral analysis

For each subject, we calculated mean subjective utility of 0, 20, and 40 points, with subjective utility being determined by satisfaction ratings on the visual analog scale after each outcome. As a measure of the concavity of the subjective utility curve for each subject, we calculated the relative subjective utility of 20 points using the following formula: 40 × (mean utility of 20 − mean utility of 0)/(mean utility of 40 − mean utility of 0), as described in our prior study [12]. The ratio of the difference in utility between 20 points and 0 points and the difference in utility between 40 points and 0 points measures the marginal utility of the first 20 points relative to the marginal utility of the entire 40 points, which will be 50% if the subjective utility curve is linear and greater than 50% if the subjective utility curve is concave. This value is then multiplied by 40 so that the result can be interpreted on the same scale as the original point scale.

To assess risk preferences for each subject, we calculated a measure of risk seeking as follows: on trials in which subjects chose between low- and high-risk gambles, we calculated the percentage of trials on which they chose the high-risk gamble. To test the hypotheses that concavity of the subjective utility curve, but not trait anxiety, would be associated with risk aversion, we performed Pearson correlation tests between risk seeking and either relative utility of 20 or STAI-T score.

As a measure of counterfactual sensitivity, for each subject we calculated the difference in mean subjective utility of 20 points when the counterfactual outcome was 0 points compared to when the counterfactual outcome was 40 points. To test the hypothesis that trait anxiety would not be associated with counterfactual sensitivity, we performed a Pearson correlation test between counterfactual sensitivity and STAI-T.

### Computational model

We modeled choices and reaction times on each trial using an LBA model. LBA parameters for each subject were estimated in R [15] using a maximum-likelihood fitting procedure with code adapted from [16]. For each subject, initial parameter values were chosen using heuristics from [16]. An objective function was then defined, calculating the likelihood of each RT value given a set of parameters. The parameters were then iteratively optimized using the *optim* function in R to determine the set of parameters which maximized the likelihood of the RT values observed in the data for that subject.

We fit two LBA models: a drift rate model and a response boundary model. For the drift rate model, following earlier LBA studies [11], we assigned a different drift rate to each stimulus type but used a common response threshold, upper end of the start point distribution, and non-decision time across responses for each subject. For each subject, we therefore estimated a single non-decision time, upper response boundary, and maximum starting-point. Additionally, for each subject, we estimated four drift rates, one for each of the experimental stimuli (i.e. the four different gambles). Each drift rate was estimated using 48 trials (because each stimulus was shown 48 times). For the response boundary model, we instead assigned a different upper response boundary to each stimulus type but used a common drift rate, upper end of the start point distribution, and non-decision time across responses for each subject. We compared these models using a quantile probability plot [17], in which response probabilities for each of the four stimuli are plotted on the x-axis and five reaction time quantiles (the .9, .7, .5, .3, and .1 quantiles) are plotted on the y-axis. We also performed numeric goodness of fit tests (chi-square goodness of fit test for choices and Kolmogorov-Smirnov goodness of fit test for reaction time distribution for each stimulus). Goodness of fit tests were performed in R. All subsequent analyses used the superior fitting model.

For each subject and stimulus, we calculated a normalized drift rate by dividing the drift rate for that stimulus by the mean drift rate across stimuli for that subject. To assess the influence of risk and EV on normalized drift rate, we performed a two-way within-subject ANOVA with normalized drift rate as dependent variable and risk and EV as predictors.

With LBA parameters estimated for each subject, we used the predicted reaction time density function with code adapted from [16] to predict response probabilities for each stimulus within each of the 6 pairs of stimuli (i.e. each decision condition). We then compared these predicted response probabilities to empirical response rates for each subject using Pearson correlation tests, to determine whether the LBA model with 4 drift rates per subject was able to capture choice behavior in the 6 different decision conditions.

To determine whether the LBA model could predict choices that were not used to estimate model parameters, we additionally performed an analysis in which half of trials were randomly selected to be held out from model-fitting (ensuring that half of trials featuring each stimulus pair were held out). We then used LBA parameters to predict choice probabilities for each stimulus pair, and compared these predicted response probabilities to empirical response rates in the held-out data for each subject, using Pearson correlation tests.

## Results

### Behavioral results

**Fig 1:**
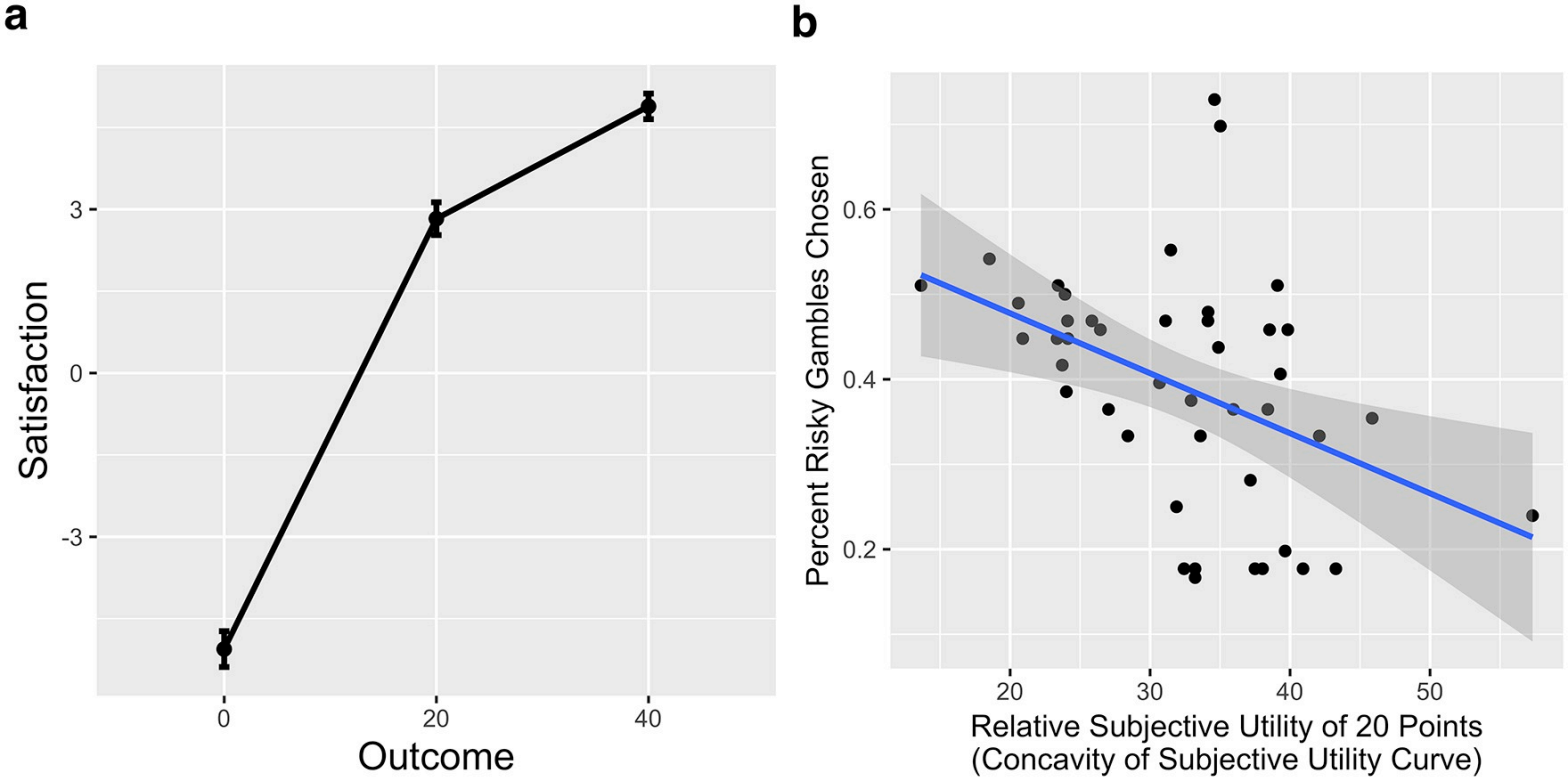
Subjective satisfaction and risk preferences. (a) Subjective utility curve constructed with mean satisfaction ratings for winning 0, 20, or 40 virtual dollars. The subjective utility curve was concave, i.e. the satisfaction with winning 20 points was greater than the mean satisfaction with winning 0 and 40 points, as predicted by economic theory. (b) Relationship between the shape of the subjective utility curve and risk preferences on the task. Subjects with a more concave subjective utility curve (i.e. a higher relative evaluation of 20 points), were more risk averse, as predicted by economic theory.

Consistent with our hypotheses, the relative subjective utility of 20 was negatively associated with risk seeking (r = -.43, p = 0.004; Fig 1). STAI-T was not associated with risk seeking (r = .11, p = 0.50).

STAI-T was not associated with counterfactual sensitivity (r = .03, p = 0.85).

### LBA model comparison

**Fig 2:**
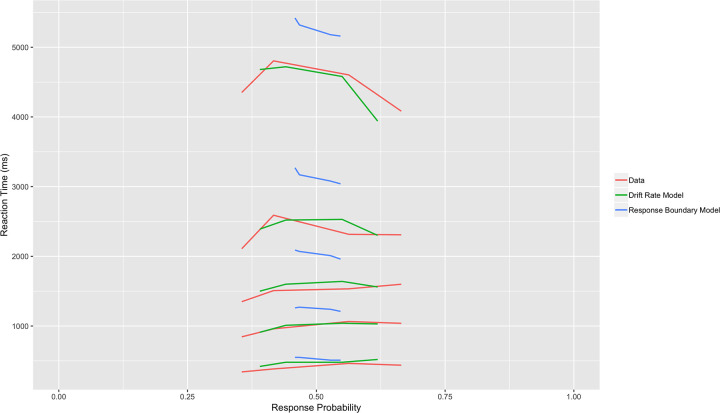
Drift rate vs. response boundary linear ballistic accumulator (LBA) models. Quantile probability plot comparing data and predictions from the drift rate and response boundary LBA models. Response probabilities for the four experimental stimuli are plotted on the x-axis, while five reaction time quantiles (the .9, .7, .5, .3, and .1 quantiles) are plotted on the y-axis. The drift rate LBA model achieved a markedly superior fit.

The quantile probability functions of the subject data, the drift rate LBA model, and the response boundary LBA model are shown in Fig 2. The drift rate LBA model (in which separate drift rates are fit for each stimulus) achieved a markedly superior fit compared to the response boundary LBA model (with both models using an equal number of parameters).

For the drift rate LBA model, chi-square goodness of fit test for choices revealed that subject behavior was significantly less random than the model’s predictions ((X^2^_3_ = 14.27, p = 0.003). Kolmogorov-Smirnov goodness of fit tests revealed good fits for reaction times for each stimulus (high risk, high EV: D = 0.05, p = 0.17; high risk, low EV: D = 0.05, p = 0.30; low risk, high EV: D = 0.03, p = 0.55; low risk, low EV: D = 0.04, p = 0.33).

For the response boundary LBA model, chi-square goodness of fit test for choices revealed that subject behavior was significantly less random than the model’s predictions ((X^2^_3_ = 130.91, p < .00001). Kolmogorov-Smirnov goodness of fit tests revealed poor fits for reaction times for each stimulus (high risk, high EV: D = 0.14, p < .00001; high risk, low EV: D = 0.20, p < .00001; low risk, high EV: D = 0.14, p < .00001; low risk, low EV: D = 0.13, p < .00001).

### LBA parameters

Mean maximum starting-point was 2129 (sd 3021). Mean non-decision time was 120 ms (sd 254 ms). Mean upper decision threshold was 2513 (sd 3069).

### Influence of risk and EV on drift rate parameters

**Fig 3:**
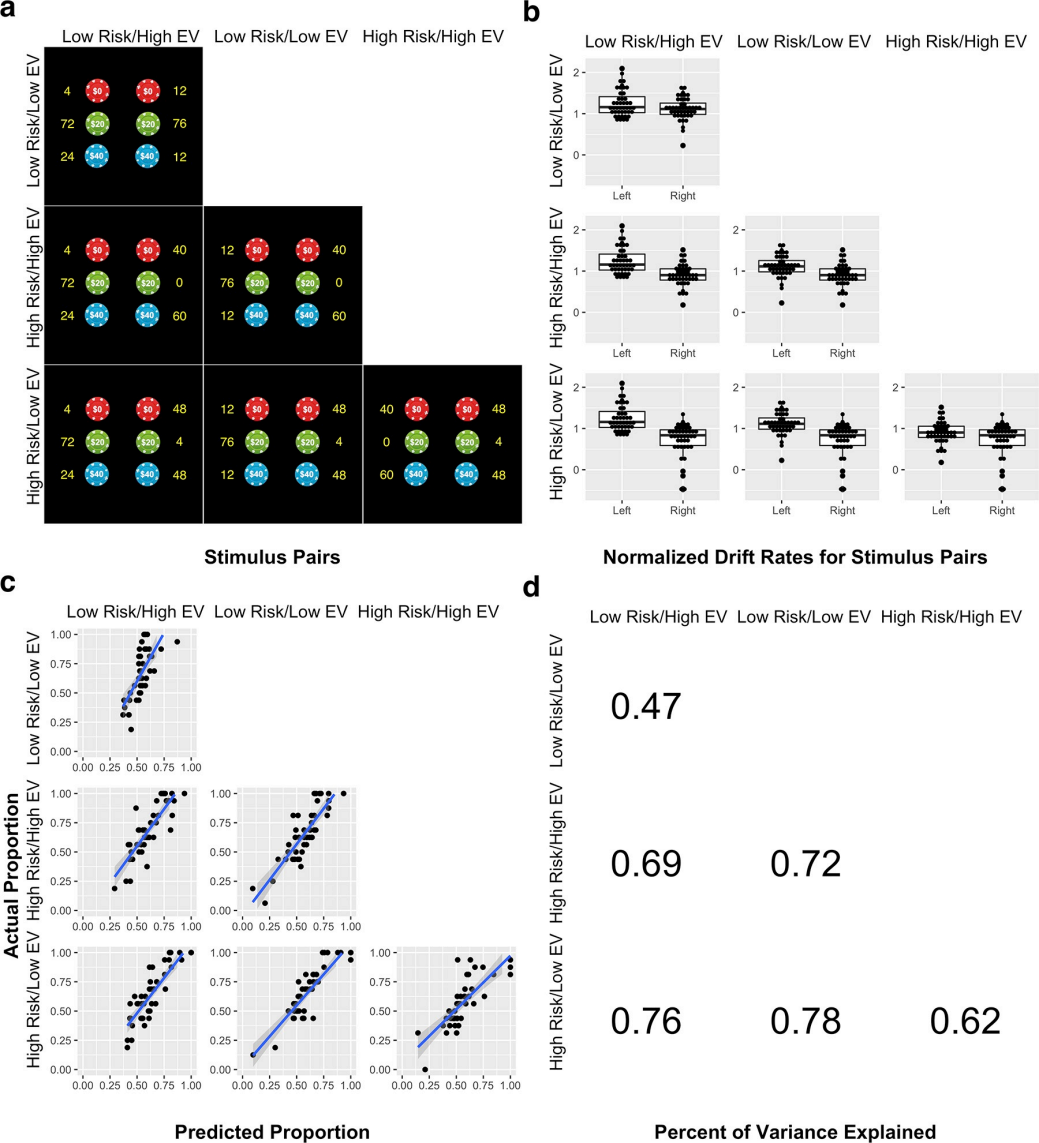
Linear ballistic accumulator (LBA) model of probabilistic decision-making. (a) The six stimulus pairs encountered by subjects in the decision-making experiment. Subjects were asked to choose one of two gambles, each of which represented a random drawing of a chip worth 0, 20, or 40 virtual dollars, from a pool of 100 chips. The yellow numbers represent the number of each chip that comprise the 100 total chips. The four decision stimuli are high risk/high EV, high risk/low EV, low risk/high EV, and low risk/low EV. (b) Normalized drift rate parameters from LBA model. Drift rates represent the average rate of accumulation of evidence for each stimulus; when evidence for a given stimulus reaches a decision threshold, that stimulus is selected. Lower risk and higher expected value (EV) were associated with higher drift rates. Subject-level drift rates are shown for each stimulus for each of the 6 possible stimulus pairs. (c) Predicted and actual choice rates for each stimulus pair when the LBA was fit using the entire dataset. Choices predicted based on LBA parameters were strongly correlated with actual subject choices for each of the six stimulus pairs. (d) Percent of variance in subject choices explained by model-based predictions for each possible stimulus pair.

There was a significant main effect of risk in predicting normalized drift rate, such that drift rates were higher for low risk stimuli (Fig 3; partial η^2^ = 0.17, F_1_ = 35.61, p < .00001). There was also a significant main effect of EV, such that drift rates were higher for high EV stimuli (partial η^2^ = 0.02, F_1_ = 4.23, p = .04). The interaction between risk and EV was not significant (partial η^2^ = 0.00, F_1_ = 0.21, p = .64).

### Comparison between model-predicted and actual choices

Predicted choices were strongly correlated with actual choices across subjects for all 6 stimulus pairs (Fig 3; high risk/low EV vs. low risk/high EV: r = .87, p < .00001; high risk/high EV vs. low risk/high EV: r = .83, p < .00001; low risk/low EV vs. low risk/high EV: r = .69, p < .00001; high risk/high EV vs. low risk/low EV: r = .85, p < .00001; high risk/low EV vs. low risk/low EV: r = .88, p < .00001; high risk/low EV vs. high risk/high EV: r = .79, p < .00001).

### Model prediction of held-out choices

When model-fitting was performed using half of trials, and then fitted parameters were used to predict choices on the remaining half of trials, predicted choices remained correlated with actual choices across subjects for all 6 stimulus pairs (high risk/low EV vs. low risk/high EV: r = .67, p < .00001; high risk/high EV vs. low risk/high EV: r = .53, p < .0002; low risk/low EV vs. low risk/high EV: r = .51, p < .0004; high risk/high EV vs. low risk/low EV: r = .61, p = .00001; high risk/low EV vs. low risk/low EV: r = .57, p = .00005; high risk/low EV vs. high risk/high EV: r = .45, p = .002).

## Discussion

The goal of this investigation was to determine whether the LBA, a sequential sampling model, can account for decision-making on an explicit probabilistic task. We examined whether the LBA could account for individual preferences based on risk and EV as well as decisional randomness (the extent to which subjects will sometimes choose an option that does not comport with their preferences, even when this choice is strictly inferior to an alternative option). We found that LBA drift rates reflect preferences for low risk and high EV options, and that predicted responses based on LBA parameters were strongly correlated with actual responses in all 6 stimulus pairs in the task. This was also the case when LBA parameters were used to predict responses on trials that were held-out (i.e. not used to estimate parameters). Assessment of model fits revealed that subject reaction times were fit well by the LBA model, while subject choices were qualitatively captured by the model, but were significantly less random than model predictions. Additionally, we replicated our earlier findings that the concavity of the subjective utility curve predicts risk preferences and that trait anxiety is unrelated to risk preferences and counterfactual sensitivity.

Our data add to the literature on irrational decision-making in general and decisional randomness in particular by demonstrating that almost all subjects sometimes chose a strictly inferior option (i.e. lower EV but the same risk), even on a task in which EV and risk are explicitly signaled to the subject. Unlike in prior studies which used a reinforcement learning context [6–8], on our task there was no explicit learning component. Subjects therefore did not gain information by choosing a low value option, and could not have been using directional exploration.

Randomness in value-based decision-making has been an important focus in the reinforcement learning literature [8]. Because reinforcement learning models themselves are not inherently random, randomness is frequently introduced via softmax decision policies. By contrast, sequential sampling models of decision-making (i.e. models of real-time evidence accumulation) inherently exhibit randomness by explicitly incorporating noise into the decision-making mechanism, and these models have been applied to decision-making studies in a number of domains. The recently proposed hypothesis that sequential sampling is the mechanism by which information about the value of different options (acquired by reinforcement learning) [10] thus offers a natural and mechanistic explanation for the randomness observed in reinforcement learning studies, even when this randomness is not rational according to economic theory. Our results extend this idea to a paradigm in which value and risk are explicitly signaled to the subject, and therefore choosing a less valuable option does not result in valuable information. While the LBA has been applied successfully to decision-making in several domains, to our knowledge, our application of the LBA to our paradigm, in which both expected value and risk are explicitly signaled to the subject, is novel. Additionally, our results help elucidate the mechanism of value-based choice in this task by strongly supporting a model in which value influences decisions via the drift rate rather than the response boundary.

A proposed explanation for observed decisional randomness is that decisions are implemented in the brain by continuously accumulating evidence in favor of each alternative, and when the evidence in favor of one alternative reaches a threshold value, a decision is reached. In this parallel accumulator model, the average rate of accumulation of evidence (i.e. drift rate) will be faster for a generally preferred option (e.g. one with lower risk and higher EV). However, due to noise in internal representations and processes, sometimes the generally less preferred option will reach the threshold first and will be selected. In the LBA model, this noise occurs in the form of variability in drift rate and in the starting point for evidence accumulation. This can occur even when the option is strictly inferior according to economic theory. Our results demonstrate that an LBA model of decision-making can qualitatively account for rates of choosing specific options across subjects, including these strictly inferior choices.

The LBA has previously been shown to account for a variety of empirically observed phenomenon in decision-making studies. This includes the speed accuracy tradeoff in response deadline paradigms, complex interactions of response times with accuracy and with experimental conditions that emphasize either speed or accuracy, and the shape of response time distributions in paradigms with more than two decision options [11], as well as context effects in multi-alternative choice [18]. The model has been applied to a variety of decision-making paradigms, including lexical tasks, visual perception tasks, choices between hedonic stimuli, and intertemporal choice [11, 18, 19], This flexibility, coupled with its relative simplicity, is a major advantage of the LBA. Conceptually, there are two sources of randomness in the LBA: (1) trial-by-trial variability in drift rates, which are drawn from a normal distribution, and (2) variability in the starting point for evidence accumulation, which is drawn from a uniform distribution from zero to a maximum value specified by one of the LBA parameters. When speed is emphasized over accuracy, this maximum value is near the decision threshold, meaning that responses can be made with very little evidence accumulation [11]. When accuracy is emphasized, the maximum starting point is far from the decision threshold; in this case, errors can still occur, due to variability in drift rates rather than starting points. Future research can examine these sources of randomness in our explicit probabilistic task in more detail.

Consistent with our prior findings [12], we found that trait anxiety is not associated with risk preferences on our decision-making task or with counterfactual sensitivity. Unlike other tasks on which risk aversion has been found in anxious subjects [20], our task only allows for gains or no gain, and does not allow for the possibility of losses. Differences in risk behavior in anxious individuals may therefore only emerge when losses are possible. Anxious subjects have also shown differences in behavior on reinforcement learning tasks, on which there is a need to adapt learning based on the statistical properties of the environment [21, 22]. Applying the LBA model to a task that includes loss outcomes or the need to adjust rates of learning could therefore further elucidate dysfunctional decision-making in anxious subjects. It is important to note that this null finding must be interpreted with caution given the relatively small sample size.

While the present study shows that an LBA model is qualitatively consistent with choice behavior and quantitatively consistent with reaction times on our task, it is likely that this model will not be sufficient to fully encapsulate decision-making. In particular, there is evidence that decision-making can be slowed to improve accuracy when there are multiple competing responses, an effect that our model does not account for [23]. The interaction between decision options may explain why subject choices were significantly less random than LBA model predictions. Another limitation of this study is the relatively small sample size, which would not allow us to detect a small effect of trait anxiety on behavior on the task. Additional limitations include the cross-sectional nature of the study and the lack of neural data to elucidate the brain circuitry underlying decisional randomness on this task. Finally, the relatively small number of trials may limit the reliability of parameter recovery, although the prediction of choices on held-out trials demonstrates that drift rates were estimated with some reliability.

In conclusion, decisional randomness is an important component of human decision-making, which can be rational or irrational according to context. We found that humans display decisional randomness even when risk and EV are explicitly signaled to the subjects. We further found that subjects’ decisions on this task, including their irrational decisions, can be captured by the LBA sequential sampling model. These findings have potential implications for other decision-making contexts and for assessing and treating disordered decision-making in psychiatric populations.

## Supporting information

S1 Table (DOCX)
