# Supplementary material for: A parallel accumulator model accounts for decision randomness when deciding on risky prospects with different expected value
Source: PLoS One. 2020 Jul 23;15(7):e0233761. doi: 10.1371/journal.pone.0233761 (PMC7377428; doi:10.1371/journal.pone.0233761)
Supplement: S1 Table — (DOCX) [file pone.0233761.s001.docx]

Supplementary Table 1

| **Subject** | **Maximum Starting-Point** | **Non-Decision Time** | **Upper Decision Threshold** |
| --- | --- | --- | --- |
| 1 | 1235.08 | 2.45 | 1952.48 |
| 2 | 26.88 | 131.83 | 1158.24 |
| 3 | 2035.85 | 122.44 | 2124.63 |
| 4 | 1364.12 | 0 | 1726.79 |
| 5 | 5680.66 | 667.81 | 5694.83 |
| 6 | 5708.57 | 0.48 | 7309.32 |
| 7 | 21735.94 | 441.36 | 26304.59 |
| 8 | 8458.82 | 605.44 | 8458.83 |
| 9 | 2420.21 | 9.41 | 4438.76 |
| 10 | 1235.58 | 738.76 | 1363.87 |
| 11 | 15330.62 | 1085.01 | 15331.12 |
| 12 | 624.48 | 171.83 | 624.96 |
| 13 | 691.78 | 536.75 | 697.62 |
| 14 | 1275.72 | 291.18 | 1293.58 |
| 15 | 89.28 | 10.07 | 89.38 |
| 16 | 497.71 | 216.9 | 497.87 |
| 17 | 1182.86 | 0.16 | 1666.44 |
| 18 | 870.91 | 188.1 | 918.16 |
| 19 | 3842.97 | 0.32 | 4268.29 |
| 20 | 3055.69 | 588.39 | 3212.94 |
| 21 | 4123.88 | 369.53 | 4545.41 |
| 22 | 51.08 | 4.38 | 51.11 |
| 23 | 834.77 | 823.63 | 838.9 |
| 24 | 43279.43 | 437.71 | 44991.93 |
| 25 | 2703.87 | 0.01 | 3068.81 |
| 26 | 1529.61 | 310.93 | 1529.61 |
| 27 | 11287.99 | 1249.83 | 12125.36 |
| 28 | 764.25 | 0.36 | 1437.76 |
| 29 | 2443.42 | 0.01 | 2643.32 |
| 30 | 659.01 | 0.21 | 736.42 |
| 31 | 1270.03 | 846.05 | 1270.98 |
| 32 | 1283.9 | 0 | 2129.08 |
| 33 | 757.43 | 169.11 | 757.43 |
| 34 | 482.72 | 345.24 | 482.72 |
| 35 | 799.25 | 0.07 | 1006.4 |
| 36 | 2529.96 | 382.3 | 3192.37 |
| 37 | 976.51 | 0 | 1311.22 |
| 38 | 0.26 | 0.01 | 304.49 |
| 39 | 1835.78 | 0.07 | 2636.82 |
| 40 | 226.34 | 0.43 | 386.55 |
| 41 | 3972.39 | 51.32 | 3972.39 |
| 42 | 647.49 | 188.67 | 657.12 |
| 43 | 761.71 | 32.05 | 1501.1 |
| 44 | 1.28 | 94.21 | 524.75 |

Individual LBA parameter values.
